# Formononetin ameliorates muscle atrophy by regulating myostatin‐mediated PI3K/Akt/FoxO3a pathway and satellite cell function in chronic kidney disease

**DOI:** 10.1111/jcmm.16238

**Published:** 2021-01-06

**Authors:** Lingyu Liu, Rong Hu, Haiyan You, Jingjing Li, Yangyang Liu, Qiang Li, Xiaohui Wu, Jiawen Huang, Xiangsheng Cai, Mingqing Wang, Lianbo Wei

**Affiliations:** ^1^ Shenzhen Hospital Southern Medical University Shenzhen China; ^2^ School of Traditional Chinese Medicine Southern Medical University Guangzhou China; ^3^ Institute of Biotherapy Southern Medical University Guangzhou China; ^4^ Huangpu People's Hospital of Zhongshan Zhongshan China; ^5^ Center for Medical Experiments University of Chinese Academy of Science‐Shenzhen Hospital Shenzhen China

**Keywords:** formononetin, inflammation, muscle atrophy, myostatin, PI3K/Akt/FoxO3a, satellite cell function

## Abstract

Muscle atrophy is a common complication in chronic kidney disease (CKD). Inflammation and myostatin play important roles in CKD muscle atrophy. Formononetin (FMN), which is a major bioactive isoflavone compound in *Astragalus membranaceus*, exerts anti‐inflammatory effects and the promotion of myogenic differentiation. Our study is based on myostatin to explore the effects and mechanisms of FMN in relation to CKD muscle atrophy. In this study, CKD rats and tumour necrosis factor α (TNF‐α)‐induced C2C12 myotubes were used for in vivo and in vitro models of muscle atrophy. The results showed that FMN significantly improved the renal function, nutritional status and inflammatory markers in CKD rats. Values for bodyweight, weight of tibialis anterior and gastrocnemius muscles, and cross‐sectional area (CSA) of skeletal muscles were significantly larger in the FMN treatment rats. Furthermore, FMN significantly suppressed the expressions of MuRF‐1, MAFbx and myostatin in the muscles of CKD rats and the TNF‐α‐induced C2C12 myotubes. Importantly, FMN significantly increased the phosphorylation of PI3K, Akt, and FoxO3a and the expressions of the myogenic proliferation and differentiation markers, myogenic differentiation factor D (MyoD) and myogenin in muscles of CKD rats and the C2C12 myotubes. Similar results were observed in TNF‐α‐induced C2C12 myotubes transfected with myostatin‐small interfering RNA (si‐myostatin). Notably, myostatin overexpression plasmid (myostatin OE) abolished the effect of FMN on the phosphorylation of the PI3K/Akt/FoxO3a pathway and the expressions of MyoD and myogenin. Our findings suggest that FMN ameliorates muscle atrophy related to myostatin‐mediated PI3K/Akt/FoxO3a pathway and satellite cell function.

## 1 INTRODUCTION

Muscle atrophy is the main pathophysiological consequence and the most effective marker of protein‐energy wasting, which is a well‐established complication in individuals with chronic kidney disease (CKD).^1^ Muscle atrophy is closely related to aggravated occurrence of other complications, such as infection and multiple organ failure.^2^ It affects patients' quality of life and can result in increased morbidity and mortality. In recent decades, study of the mechanisms of CKD muscle atrophy has made substantial progress. However, no reliably effective pharmacological or therapeutic approaches to prevent CKD‐induced muscle atrophy currently exist.^3^ In‐depth study of the molecular mechanisms of CKD muscle atrophy and the search for effective drugs for the treatment of CKD muscle atrophy are of major concern.

Inflammation plays a critical role in the pathogenesis and progression of CKD muscle atrophy. In CKD, chronic inflammation can lead to poor appetite, reduced protein and calorie intake, and decreased protein synthesis, further leading to protein‐energy expenditure and hypoproteinemia.^4^, ^5^ In this condition, increased levels of circulating inflammatory cytokines, including tumour necrosis factor α (TNF‐α), Interleukin‐6 (IL‐6), Interleukin‐8 (IL‐1β) and amyloid A, stimulate muscle catabolism.^6^, ^7^ The catabolic pathways triggered by inflammation that leading to protein wasting include the activation of the ubiquitin‐proteasome system (UPS), the autophagy‐lysosome systems, caspase‐3 and myostatin.^8^, ^9^, ^10^ Most importantly, inflammatory cytokines, particularly TNF‐α, can activate myostatin, which accelerates protein catabolism and leads to skeletal muscle atrophy.^11^, ^12^, ^13^ Hence, inhibiting the production of inflammatory cytokines and then inhibiting the expression of myostatin may be an effective means of treating CKD muscle atrophy.

Myostatin, a member of the transforming growth factor beta (TGF‐β) superfamily, is a negative regulator of skeletal muscle mass and is closely associated with protein degradation and the inhibition of the proliferation and differentiation function of satellite cells. It is expressed predominantly in skeletal muscle. It negatively regulates the activation of satellite cells, controls the self‐renewal process, and inhibits myoblast proliferation and differentiation.^14^, ^15^, ^16^ A number of conditions, including CKD, hindlimb unloading, disuse atrophy, can lead to increased myostatin expression.^17^, ^18^, ^19^, ^20^ In addition, gene deletion and naturally occurring mutations in myostatin induce muscle fiber hypertrophy and an increase in muscle mass and bodyweight, and the overexpression of myostatin decreases fiber size and muscle mass.^21^, ^22^, ^23^, ^24^ There is evidence that the subcutaneous injection of anti‐myostatin peptibody can block protein degradation through the PI3K/Akt/FoxO3a pathway and improve the function of satellite cells in a CKD mouse model.^12^ Finally, a phase I/II clinical trial of the myostatin‐neutralizing antibody MYO‐029 showed that administration of myostatin inhibitors had good safety and tolerability and increased muscle size.^25^

Formononetin (FMN) is a bioactive isoflavone compound appeared in Chinese medicinal ingredients, such as *Astragalus membranaceus*. Recent research has found that FMN has multiple pharmacological effects, including anti‐inflammatory, antioxidant, antitumor and vasorelaxant benefits.^26^, ^27^, ^28^ Several studies have demonstrated the anti‐inflammatory action of FMN. One showed that FMN down‐regulated TNF‐α, IL‐6 and IL‐1β in a lipopolysaccharide (LPS)‐induced zebrafish with lipidomics and targeted transcriptomics.^29^ Another found that FMN ameliorated mast cell‐mediated allergic inflammation via reducing the production of the pro‐inflammatory cytokine TNF‐α, IL‐1β and IL‐6 by inhibiting NF‐κB activation and upstream IκKα phosphorylation, as well as inhibiting caspase‐1 activity.^30^ In addition, FMN attenuates LPS‐induced inflammation in mice associated with the induction of PPAR gamma expression.^31^ Furthermore, FMN exhibits anti‐inflammatory actions in methotrexate‐induced rats by up‐regulating nuclear factor erythroid 2‐related factor (Nrf2) / hemeoxygenase‐1 (HO‐1) signalling.^32^ Notably, a recent study showed that FMN significantly increased myogenic markers such as MyoD and myogenin in C2C12 myoblast.^33^ However, little is known about the effects of FMN in CKD muscle atrophy.

In the current study, based on the myostatin‐mediated PI3K‐Akt‐FoxO3a pathway and the proliferation and differentiation function of satellite cells, we investigated the role and mechanisms of FMN in CKD muscle atrophy in 5/6 nephrectomy rats in vivo and a TNF‐α‐induced C2C12 in vitro myotubes model.

## 2 MATERIALS AND METHODS

### 2.1 Chemicals and reagents

FMN was purchased from Dalian Meilun biotechnology co., Ltd. (CAS 485‐72‐3; molecular formula: C16H12O4; molecular weight: 268.27; purity >98%, Dalian, China). Recombinant murine TNF‐α was purchased from Pepro Tech (Catalog Number: 315‐01A; USA).

### 2.2 Experimental animals and experimental design

**FIGURE 1:**
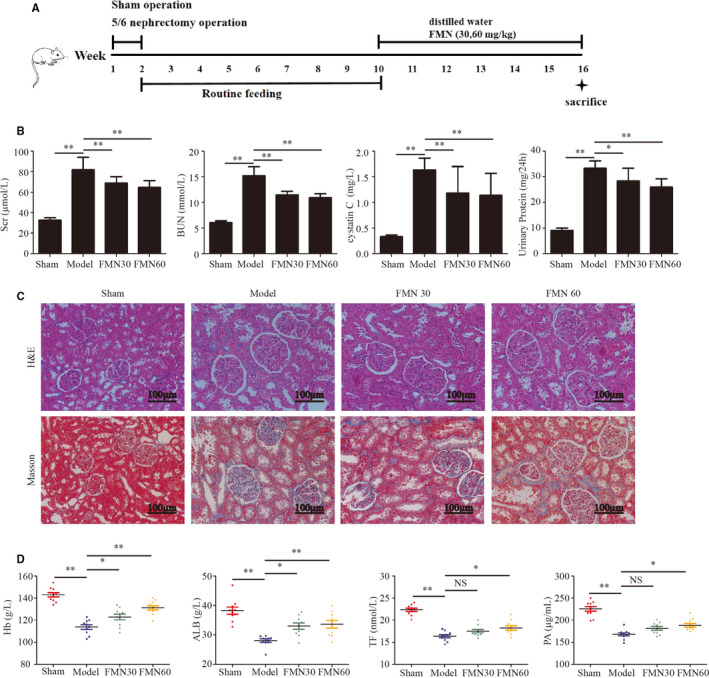
FMN improves renal function and kidney pathology, alleviates inflammation and ameliorates the nutrition index in CKD rats. Two‐step subtotal nephrectomy was performed on 8‐wk‐old male SD rats to induce muscle atrophy, and they were administered FMN (30 and 60 mg/kg) daily for 6 wk. After this, they were sacrificed (n = 10/group). (A) Schematic protocol for the animal experiment. (B) Renal function indexes, including BUN, creatinine, cystatin C and 24 h urinary protein (n = 10/group). (C) Paraffin sections from kidney tissues stained with H&E and Masson and observed under a microscope (magnification ×200). (D) The nutrition indexes of Hb, ALB, TF and PA in each group (n = 10/group). Significant differences are indicated as *P < .05, **P < .01 compared with the sham group or the CKD model group

All animal experiments and procedures were approved by the Institutional Animal Care and Use Committee of Southern Medical University. Male Sprague‐Dawley (SD) rats weighing approximately 200 ± 20 *g* purchased from the Experimental Animal Centre of Southern Medical University (permitted by SCXK 2016‐0041 [Guangzhou]). The animals were housed in a specific‐pathogen‐free animal laboratory. After a 1‐week acclimatization period, the animals were randomly assigned to the sham operation or the 5/6 nephrectomy group. Each animal in the nephrectomy group underwent subtotal nephrectomy in two stages as described previously.^10^, ^12^ During the first stage, approximately two‐thirds of the left kidney was removed after ligation of both the upper and lower poles. The entire right kidney was removed 1 week later, after the ligation of the vascular pedicle. Sham‐operated rats underwent the same procedure but without the surgical reduction of the kidney. The procedures were performed under chloral hydrate anaesthesia, using a strict aseptic technique. After the 5/6 nephrectomy and 8 weeks of routine feeding, measurement of serum levels of creatinine (Scr) and blood levels of urea nitrogen (BUN) was done to determine whether the animal model had been successfully established. The 5/6 nephrectomy animals were randomly divided into three groups (n = 10): CKD model group, CKD + FMN 30 mg/kg group and CKD + FMN 60 mg/kg group. FMN (30 and 60 mg/kg/d) was administered by oral gavage in the form of a suspension for 6 weeks. The sham operation group and the CKD model group were given an equal volume of distilled water. The experimental procedure is presented in Figure 1A.

### 2.3 Specimen collection

After the completion of the treatment, each animal was kept in a metabolic cage for 24 hours to collect 24 hours urine samples. Blood samples were collected and then centrifuged at 3000 *g* for 15 minutes to obtain serum. The tibialis anterior and gastrocnemius muscles were harvested and weighed immediately after they were taken from the carcass. The kidney tissues were dissected immediately and fixed with 4% paraformaldehyde.

### 2.4 Biochemical parameters

The levels of Scr, BUN, cystatin C, 24 hours urinary protein, haemoglobin (Hb), albumin (ALB), transferrin (TF) and prealbumin (PA) were measured using the Hitachi Model 7100 Automatic Analyzer (Hitachi, Japan), according to the manufacturer's instructions (Nanjing Jiancheng Bioengineering Institute, China).

### 2.5 Haematoxylin and eosin staining

The tibialis anterior muscle tissues and kidney tissues were fixed in 4% paraformaldehyde for 48 hours and embedded in paraffin after dehydration. These samples were sliced into 4 mm sections, dewaxed with xylene, rehydrated in a graded ethanol series to water and finally stained with haematoxylin and eosin (H & E). Four images were taken randomly for each sample, and the average muscle fiber cross‐sectional area (CSA) was assessed using the Image J software. The pathological morphology of the kidney tissues was observed under a microscope.

### 2.6 Masson's trichrome staining

Paraffin‐embedded kidney tissues were sliced into 4 mm sections, deparaffinized and stained with haematoxylin solution for 10 minutes, washed in running water for 10 minutes. Then, the sections were stained in Biebrich scarlet‐acid fuchsin solution for 10 minutes, rinsed in distilled water and differentiated in phosphomolybdic‐phosphotungstic acid solution for 10 minutes. Next, they were placed in aniline blue solution for 10 minutes, rinsed in distilled water and differentiated in 1% acetic acid solution for 5 minutes. Finally, the sections were washed in distilled water and dehydrated in gradient ethanol solutions, cleaned and imaged under a microscope.

### 2.7 Cell culture and treatments

Mouse C2C12 myoblasts were purchased from the Type Culture Collection of the Chinese Academy of Sciences (Shanghai, China). C2C12 myoblasts were cultured in high‐glucose DMEM (Gibco, USA) and supplemented with 10% foetal bovine serum (Gibco, New York, USA) and 1% penicillin‐streptomycin (Gibco, New York, USA) at 37°C in a humidified atmosphere of 5% CO_2_. When the myoblasts were at 80% confluence, the culture medium was replaced with a differentiation medium (DMEM supplemented with 2% horse serum) for another 5 days to induce differentiation and to form myotubes. To explore the signal transduction pathways involved in the effects of FMN on muscle atrophy, the C2C12 myotubes were treated with FMN (25 or 50 μmol/L) with or without TNF‐α (40 ng/mL) for another 48 hours.

### 2.8 Cell viability assay

The cytotoxicity of TNF‐α and FMN in C2C12 myotubes was measured using CCK‐8 kit. Briefly, C2C12 myoblasts were seeded into 96‐well plates (1000 cells/well) in the growth medium. When the C2C12 myoblasts reached 80% confluence, the cells were switched to grow in differentiation medium at 37°C with 5% CO_2_ for 5 days to differentiate into myotubes. The C2C12 myotubes were incubated with different concentrations of TNF‐α (0, 20, 40, 60, 80 and 100 ng/mL) for 24 hours and 48 hours and FMN (0, 5, 10, 25, 50 and 100 μmol/L) for 48 hours. Then, 10 μL CCK‐8 was added to each well for 45 minutes incubation at 37°C. The absorbance at 450 nm was measured using a microplate reader (S5 Versa Analyzer, USA). The results were averaged from five duplicate wells of each group.

### 2.9 Immunofluorescence staining

Paraffin‐embedded tibialis anterior muscles were sliced into 4 mm sections, dewaxed with xylene and rehydrated in a graded ethanol series to water. Next, antigen retrieval was performed using 0.01 mol/L Citrate Antigen Retrieval Solution (pH 6.0) (G1202, Servicebio, Wuhan, China) in microwave oven (medium fire 8 minutes, stop fire 8 minutes and turn to medium low fire for 7 minutes). After natural cooling, they were washed with PBS (pH 7.4) 3 times (each time for 5 minutes) on a shaker. Sections were circled with histological pen, quenched with autofluorescence quenching agent (G1221, servicebio) for 5 minutes and then washed with running water for 10 minutes. Then, the sections were blocked with 5% BSA for 30 minutes at room temperature. Thereafter, the sections were incubated with anti‐myostatin (1:200; Abcam, ab124721), anti‐MAFbx (1:200; Abcam, ab168372), anti‐MuRF‐1 (1:200; Abcam, ab201941), anti‐MyoD (1:200; Abcam, ab16148) and anti‐myogenin (1:200; Abcam, ab212668) at 4°C overnight incubation. The sections were washed with PBS and incubated with fluorescent secondary antibody (Thermo Fisher Scientific, USA) in dark for 1 hour at room temperature. After washed with PBS 3 times, the sections were incubated with DAPI for 10 minutes in dark room temperature. Finally, the sections were washed with PBS 3 times, sealed with Fluorescence decay‐resistant Medium (G1401, Servicebio) and placed under a fluorescence microscope to observe and collect images.

The C2C12 myotubes were fixed with 4% formaldehyde for 20 minutes. They were permeabilized with 0.25% Triton X‐100 in PBS for 10 minutes and were blocked with 5% BSA solution for 30 minutes. Then, the C2C12 myotubes were incubated with the following primary antibodies: anti‐myostatin, anti‐MAFbx and anti‐MuRF‐1 at 4°C overnight, followed by incubation with a corresponding fluorescent secondary antibody at 37°C for 1 hour. Finally, five images were taken randomly with a fluorescence microscope for each sample, and the fluorescence intensity was analysed with Image J.

### 2.10 Enzyme‐linked immunosorbent assay

The levels of CRP, TNF‐α, IL‐6 and IL‐1β in serum were evaluated using commercial enzyme‐linked immune sorbent assay (ELISA) kits (R&D Systems, USA). ELISA analyses were performed independently in duplicates for each sample, following the manufacturer's instructions.

### 2.11 Quantitative real‐time polymerase chain reaction (qPCR)

The mRNA expression levels of inflammatory markers, differentiation and muscle atrophy‐related genes in muscle tissues and C2C12 myotubes were measured using qPCR. Total RNA was isolated from muscle tissues and C2C12 myotubes using the Trizol reagent (Invitrogen Life Technologies, USA). The concentration and purity of RNA were assessed by determining the absorbance ratio at 260 nm and 280 nm and reverse transcribed into cDNA using the PrimeScriptTM 1st Strand cDNA Synthesis Kit (TaKaRa). qPCR was conducted in 20 µL reactions using a TB Green Premix Ex TaqTM (RR420A, Takara, Japan). All gene expression levels were normalized, using GAPDH as an endogenous control. The primer sequences used in the experiments are shown in Table 1.

**TABLE 1:** The list of primer sequences used in qPCR

Gene		Sequence
CRP	Sense Anti‐sense	CCTTCGTATTTCCCGGAGTGTC CTCACATCAGCGTGGGCATAG
TNF‐α	Sense Anti‐sense	CATGAGCACAGAAAGCATGATCCG AAGCAGGAATGAGAAGAGGCTGAG
IL‐6	Sense Anti‐sense	AAGCCAGAGTCATTCAGAGCAA GTCTTGGTCCTTAGCCACTCCT
IL‐1β	Sense Anti‐sense	TGATGAAAGACGGCACCC TGTCCCGACCATTGCTGTTT
Myostatin	Sense Anti‐sense	TGGCATTACTCAAAAGCAAAAAG CATCAATACTCTGCCAAATACCA
MyoD	Sense Anti‐sense	ACGACTGCTTTCTTCACCACTCCT TCGTCTTAACTTTCTGCCACTCCG
MyoG	Sense Anti‐sense	CCAGTGAATGCAACTCCCAC GCATGGTTTCGTCTGGGAAG
GAPDH	Sense Anti‐sense	ACTCCACTCACGGCAAATTCA CGCTCCTGGAAGATGGTGAT

### 2.12 Myostatin‐small interfering RNA (si‐myostatin) and myostatin overexpression plasmid (myostatin OE) transfection

The si‐myostatin and scramble siRNA (si‐NC) was obtained from Kidan Bio Technology Co., Ltd. (Guangzhou, China). The sequences of si‐myostatin and si‐NC are shown in Table 2.

**TABLE 2:** The sequences of si‐myostatin and si‐NC

Name		Sequence
si‐myostatin‐1	Sense Anti‐sense	CCCAUGAAAGACGGUACAATT UUGUACCGUCUUUCAUGGGTT
si‐myostatin‐2	Sense Anti‐sense	GGAUGAGAAUGGCCAUGAUTT AUCAUGGCCAUUCUCAUCCAA
si‐myostatin‐3	Sense Anti‐sense	CCCGUCAAGACUCCUACAATT UUGUAGGAGUCUUGACGGGTC
si‐NC	Sense Anti‐sense	GUGAGCGUCUAUAUACCAUTT AUGGUAUAUAGACGCUCACTT

The coding sequences of the myostatin gene were obtained from GenBank, and the primers (sense, 5′‐ATGATGCAAAAACTGCAAATGT‐3′; anti‐sense, 5′‐TCATGAGCACCC ACAGCG‐3′) were obtained from The Beijing Genomics Institute. After PCR amplification of the myostatin cDNA sequences, restriction enzyme digestion and DNA sequencing, we ligated the myostatin cDNA sequences and the PCDNA 3.1 (+) vector fragments by T4 lignase. Thus, the PCDNA 3.1 (+)‐myostatin plasmid (myostatin OE) was successfully constructed.

C2C12 myotubes were transfected with si‐myostatin (or si‐NC) or myostatin OE (or vehicle vector) for 24 hours using Lipofectamine 3000 (Invitrogen, Carlsbad, CA, USA) according to the manufacturer's instructions.

### 2.13 Western blotting analyses

Muscle tissues and C2C12 myotubes were lysed in RIPA buffer for 30 minutes, followed by homogenate and centrifugation at 12 000 *g* for 15 minutes at 4°C. The protein concentrations were determined using a BCA protein assay kit (Thermo Fisher Scientific, USA). The protein samples were applied to 12% SDS polyacrylamide gels, transferred to PVDF membranes, blocked with 5% BSA TBST buffer and incubated with the following primary antibodies according to the manufacturer's recommendations: anti‐myostatin, anti‐MAFbx, anti‐MuRF‐1, anti‐MyoD, anti‐myogenin (all 1:1000; Abcam, England), p‐PI3K, PI3K, p‐Akt, Akt, p‐FoxO3a, FoxO3a (all 1:1000; Affinity, USA) and GAPDH (1:10 000; Affinity, AF7021). Then, the membranes were incubated with a corresponding secondary antibody (1:5000; Affinity, USA) at room temperature for 1 hour and washed with 1× TBST 3 times. Finally, the protein bands were visualized with an ECL Western Blotting Substrate Kit (Millipore, 1622301, USA) and were captured in a CCD system (Image Station 2000 MM, Kodak, USA).

### 2.14 Statistical analyses

Statistical analysis was conducted in SPSS 22.0 software (SPSS, Chicago, USA) and GraphPad Prism Software 5.0 (CA, US). Results are expressed as the mean ± standard deviation. Data were analysed by one‐way ANOVA *P*‐value <.05 was considered statistically significant.

## 3 RESULTS

### 3.1 FMN improves renal function and nutrition index in CKD rats

Scr, BUN, Cystatin C and 24‐hour urinary protein levels in CKD model group were significantly higher than those in sham operation group. However, these levels decreased significantly after 6 weeks of FMN administration (Figure 1B). Histopathological analyses of the kidney tissues using H&E and Masson's trichrome staining revealed a basically normal kidney morphology in the sham group and significant histological irregularities, such as glomerular sclerosis, interstitial fibrosis and inflammatory cell infiltration in the CKD model group. Kidney morphology was significantly better in the FMN groups (Figure 1C). The levels of Hb, ALB, TF and PA were lower in the CKD model than in the sham group. However, the HB and ALB levels were significantly higher in both FMN administration groups, and the TF and PA levels were higher in only the FMN 60 mg/kg group (Figure 1D).

### 3.2 Formononetin ameliorates muscle atrophy in CKD rats

**FIGURE 2:**
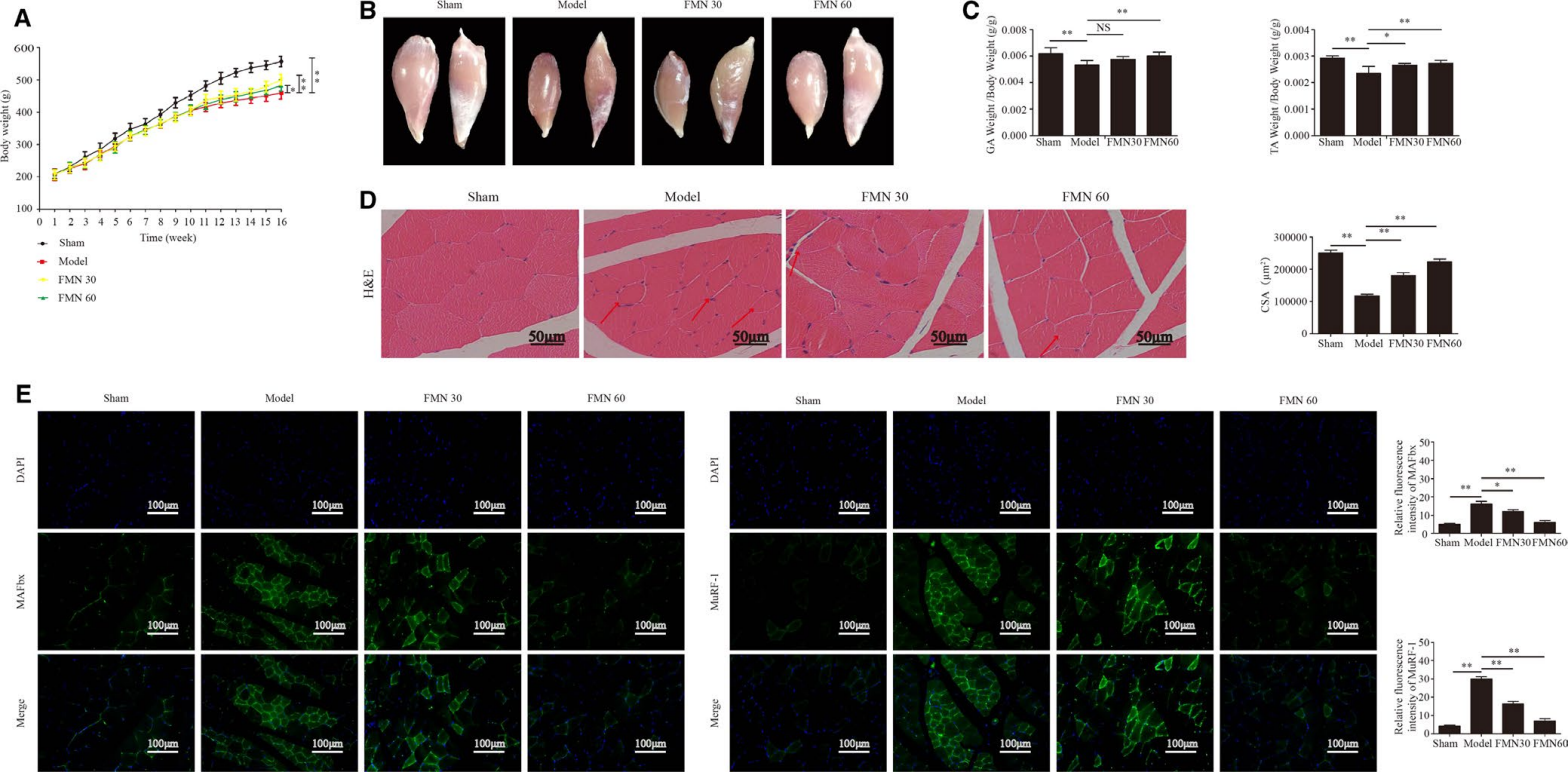
FMN significantly improves muscle atrophy in CKD rats. (A) Bodyweight was determined weekly throughout the entire 16‐wk experimental period (n = 10/group). (B) Images of gastrocnemius and tibialis anterior muscles in each group, obtained with a stereoscope. (C) The weights of the gastrocnemius and tibialis anterior muscles were measured and normalized to the final bodyweight after sacrifice (n = 6/group). (D) Paraffin sections from the tibial anterior muscle tissues stained with H&E and observed under a microscope (magnification ×400). The red arrows indicate myofibres affected by atrophy. The cross‐sectional area of the muscle fibres of different groups was measured using Image J. (E) The MAFbx and MuRF‐1 expressions in the tibialis anterior muscle determined with immunofluorescence staining (200×) using anti‐MAFbx and anti‐MuRF‐1 (green) and the nuclei detected via DAPI staining (blue). The relative fluorescence intensities of MAFbx and MuRF‐1 were compared between the groups. *P < .05, **P < .01

Bodyweight in each group increased over the course of the 16‐week experiment, and the sham group gained weight faster than the other groups. After the administration of FMN, the final bodyweight was significantly increased in the FMN administration groups relative to the CKD model group (Figure 2A).

The gastrocnemius and tibial anterior muscle volumes were significantly larger in the FMN groups than in the CKD model group (Figure 2B). The weights of the gastrocnemius and tibialis anterior muscles were measured and normalized to the final bodyweight and showed similar trends (Figure 2C). H&E staining of the tibial anterior muscle tissues showed that inflammatory cell infiltration, uneven muscle fibre thickness and cell nucleus displacement. The CSA of the muscle fibre were significantly decreased in the model group. After treatment with FMN, the above phenomena were significantly improved (Figure 2D).

The immunofluorescence staining results showed that the expressions of MAFbx and MuRF‐1 were significantly increased in tibialis anterior muscle of the CKD model group. Moreover, treatment with FMN significantly suppressed the expressions of MAFbx and MuRF‐1 (Figure 2E).

### 3.3 Formononetin ameliorates muscle atrophy induced by TNF‐α in C2C12 myotubes

**FIGURE 3:**
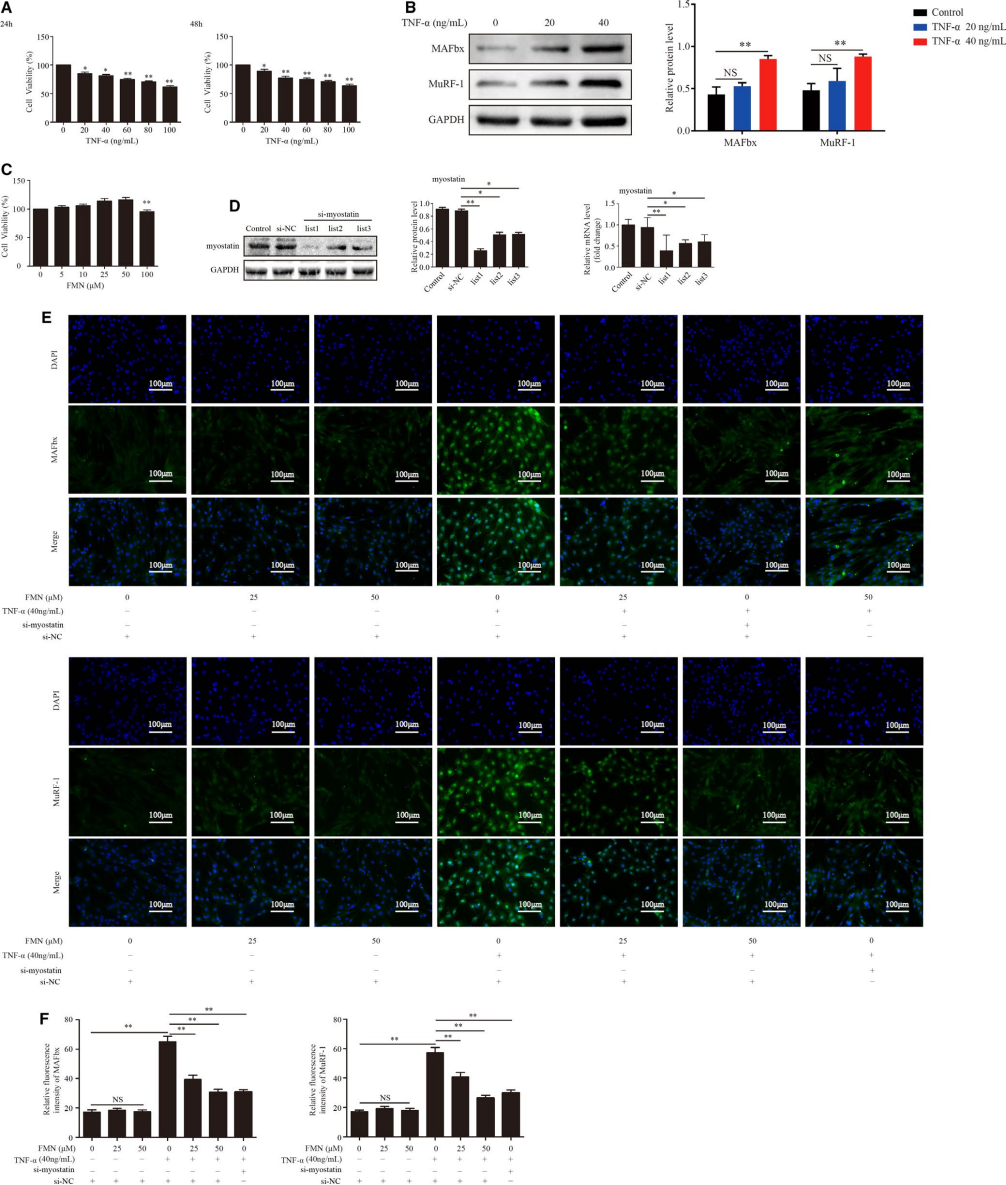
FMN ameliorates muscle atrophy induced by TNF‐α in C2C12 myotubes. (A) Cytotoxicity of TNF‐α in C2C12 myotubes. (B) The protein expressions of MAFbx and MuRF1 were detected in C2C12 myotubes treated with TNF‐α (20 or 40 ng/mL) for 48 h. (C) The cytotoxic effects of FMN assessed using a CCK‐8 assay. (D) The protein and mRNA expressions of myostatin were detected in C2C12 myotubes transfected with si‐NC or si‐myostatin. *P < .05, **P < .01 compared to the si‐NC group (n = 3/group). (E) C2C12 myotubes were treated with FMN (25 μmol/L and 50 μmol/L) in the presence or absence of TNF‐α (40 ng/mL) for 48 h following 24 h of incubation with si‐myostatin or si‐NC. The expressions of MAFbx and MuRF1 in C2C12 myotubes were determined by immunofluorescence staining (200×) with anti‐MAFbx and anti‐MuRF1 (green), and the nuclei were detected with DAPI staining (blue). (F) Relative fluorescence intensities of MAFbx and MuRF‐1 compared between groups. *P < .05, **P < .01

The results showed that treatment with 40 ng/mL TNF‐α for 48 hours significantly reduced cell viability and increased the expression of MAFbx and MuRF‐1. (Figure 3A,B). Therefore, 40 ng/mL TNF‐α incubation for 48 hours was used as atrophic model. The cytotoxicity of FMN showed that low concentrations (5, 10, 25 and 50 μmol/L) of FMN did not have cytotoxic effects, while high concentrations (100 μmol/L) induced significant cytotoxicity (Figure 3C), so we used 25 and 50 μmol/L for further experiments.

Then, we examined the role of si‐myostatin and FMN in myotube atrophy. si‐myostatin and si‐NC were transfected into C2C12 for 48 hours. The protein and mRNA expressions of myostatin were decreased after transfection (Figure 3D). The expressions of MAFbx and MuRF‐1 in the C2C12 myotubes by immunofluorescence staining showed that TNF‐α significantly increased the expressions of MAFbx and MuRF‐1. However, FMN and si‐myostatin significantly reduced the expressions of MAFbx and MuRF‐1 (Figure 3E,F).

### 3.4 FMN inhibits inflammation and the expressions of myostatin in the muscles of CKD rats and C2C12 myotubes

**FIGURE 4:**
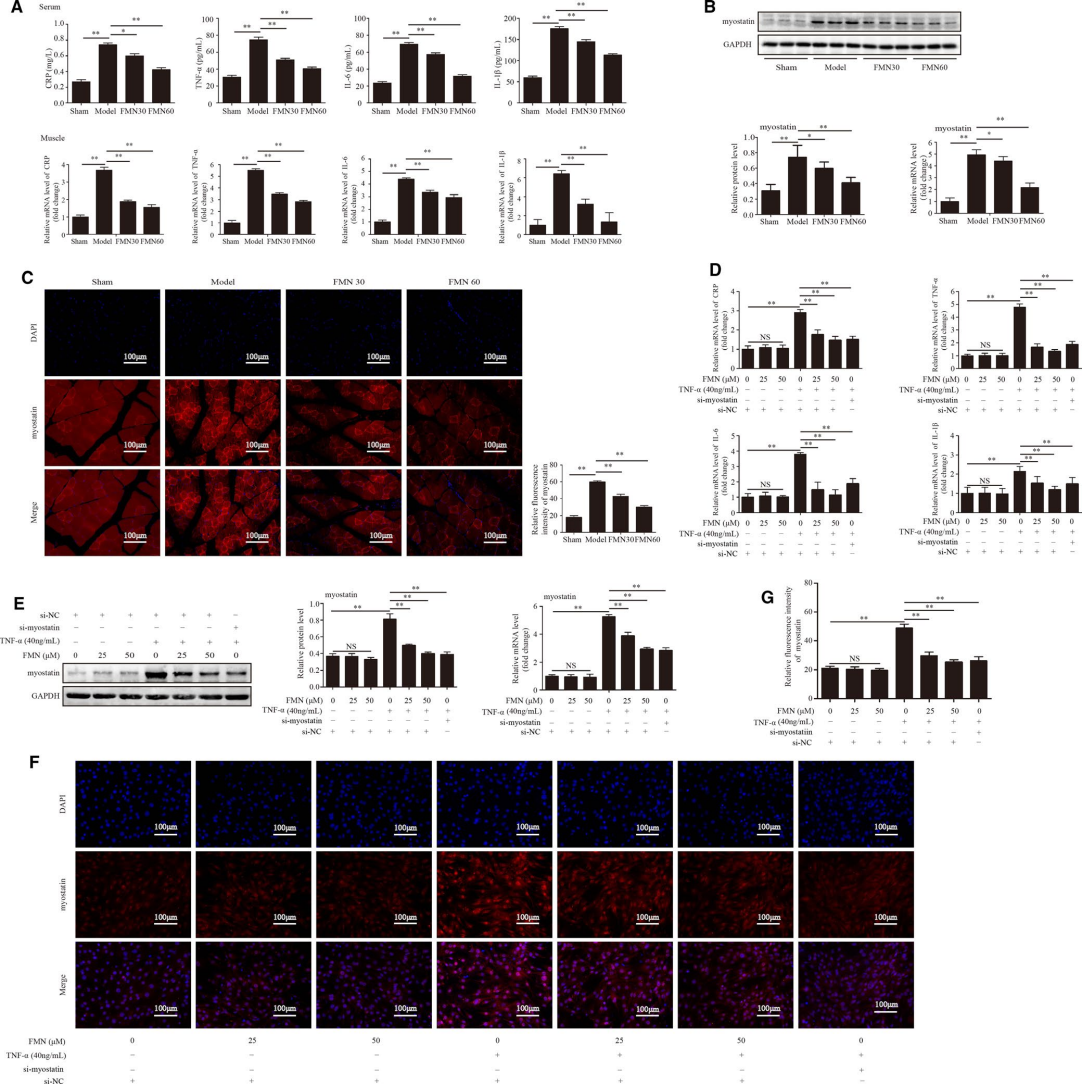
FMN suppressed the expression of inflammation and myostatin in the muscle of CKD rats and in the TNF‐α‐induced C2C12 myotubes. (A) Levels of inflammatory markers CRP, TNF‐α, IL‐6 and IL‐1β in serum and muscle were detected by ELISA (n = 8/group) and qPCR (n = 3/group). (B) The protein and mRNA levels of myostatin were analysed in the gastrocnemius muscle (n = 3/group). (C) Myostatin expression in tibialis anterior muscles determined by (200×) immunofluorescence staining with anti‐myostatin (red) and nuclei was detected by DAPI staining (blue). The relative fluorescence intensity of myostatin was compared between the groups. (D) The levels of CRP, TNF‐α, IL‐6 and IL‐1β in C2C12 myotubes were determined by qPCR (n = 3/group). (E) The protein and mRNA levels of myostatin analysed with Western blotting and qPCR (n = 3/group). (F) Myostatin expression determined with immunofluorescence staining (200×) with anti‐myostatin (red) and nuclei detected with DAPI staining (blue). (G) The relative fluorescence intensity of myostatin was compared between the groups. *P < .05, **P < .01

The levels of CRP, TNF‐α, IL‐6 and IL‐1β in serum and muscles were significantly greater in the CKD model group than in the sham group. After treatment with FMN, the levels of these were decreased (Figure 4A). The expressions of myostatin in muscle were examined by Western blotting, qPCR and immunofluorescent staining. The results showed that CKD significantly activated the expressions of myostatin. Moreover, treatment with FMN effectively suppressed the expression of myostatin (Figure 4B,C).

In vitro, the effects of FMN induced by TNF‐α (40 ng/mL) on the inflammatory markers in C2C12 myotubes were investigated. The qPCR results showed that TNF‐α significantly up‐regulated the expressions of CRP, TNF‐α, IL‐6 and IL‐1β. Moreover, FMN (25 and 50 μmol/L) and si‐myostatin significantly decreased the levels of each of these in TNF‐α‐induced C2C12 myotubes (Figure 4D).

The effect of FMN on the myostatin expression induced by TNF‐α (40 ng/mL) in C2C12 myotubes was also examined by Western blotting, qPCR and immunofluorescent staining. TNF‐α remarkably increased the expression of myostatin, but when the myotubes were incubated with FMN (25 and 50 μmol/L) and si‐myostatin, the increased expression was counteracted (Figure 4E‐G).

### 3.5 FMN inhibits myostatin‐mediated dephosphorylation of PI3K/Akt/FoxO3a in the muscles of CKD rats and C2C12 myotubes

**FIGURE 5:**
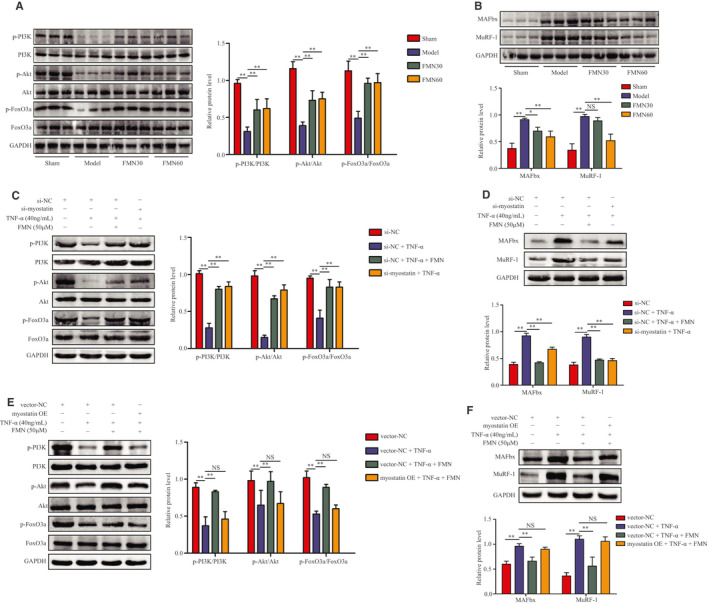
FMN inhibits myostatin‐mediated dephosphorylation on the PI3K/Akt/FoxO3a signalling pathway in the muscle of CKD rats and C2C12 myotubes. (A) Protein levels of p‐PI3K, PI3K, p‐Akt, Akt, p‐FoxO3a and FoxO3a in gastrocnemius muscle analysed using Western blotting (n = 3/group). (B) Protein levels of MAFbx and MuRF‐1 in gastrocnemius muscles analysed using Western blotting (n = 3/group). (C) C2C12 myotubes were treated with FMN (50 μmol/L) in the presence or absence of TNF‐α (40 ng/mL) for 48 h following 24 h of incubation with si‐myostatin or si‐NC. The myotubes were divided into four groups: si‐NC, si‐NC + TNF‐α, si‐NC + TNF‐α + FMN (50 μmol/L) and si‐myostatin + TNF‐α. Protein levels of p‐PI3K, PI3K, p‐Akt, Akt, p‐FoxO3a and FoxO3a in C2C12 myotubes (n = 3/group). (D) Protein levels of MAFbx and MuRF‐1 in C2C12 myotubes (n = 3/group). (E) Myostatin OE transfection was used to overexpress myostatin in C2C12 myotubes, and they were incubated with FMN (50 μmol/L) and TNF‐α for another 48 h. The myotubes were divided into four groups: vector NC, vector NC + TNF‐α, vector NC + TNF‐α + FMN (50 μmol/L) and myostatin OE + TNF‐α + FMN (50 μmol/L). The protein levels of p‐PI3K, PI3K, p‐Akt, Akt, p‐FoxO3a and FoxO3a in the myotubes were analysed using Western blotting. (F) Protein levels of MAFbx and MuRF1 in C2C12 myotubes analysed by Western blotting (n = 3/group). *P < .05, **P < .01

The result showed that the protein expressions of p‐PI3K, p‐Akt, p‐FoxO3a, MAFbx and MuRF‐1 in the muscles were significantly decreased in CKD rats, and these decreases were reversed by treatment with FMN (Figure 5A,B).

In vitro, the expressions of p‐PI3K, PI3K, p‐Akt, Akt, p‐FoxO3a and FoxO3a induced by TNF‐α in C2C12 myotubes were further examined. Western blotting results showed that FMN and si‐myostatin increased the expressions of p‐PI3K, p‐Akt and p‐FoxO3a, which were significantly decreased by TNF‐α (Figure 5C,D). In addition, the myostatin overexpression plasmid significantly blocked the change in the expressions of p‐PI3K, p‐Akt and p‐FoxO3a due to FMN. Myostatin OE also counteracted the decrease in the expressions of MAFbx and MuRF‐1 due to FMN in C2C12 myotubes (Figure 5E,F).

### 3.6 FMN improves myostatin‐mediated satellite cell proliferation and differentiation dysfunction in CKD rats and C2C12 myotubes

**FIGURE 6:**
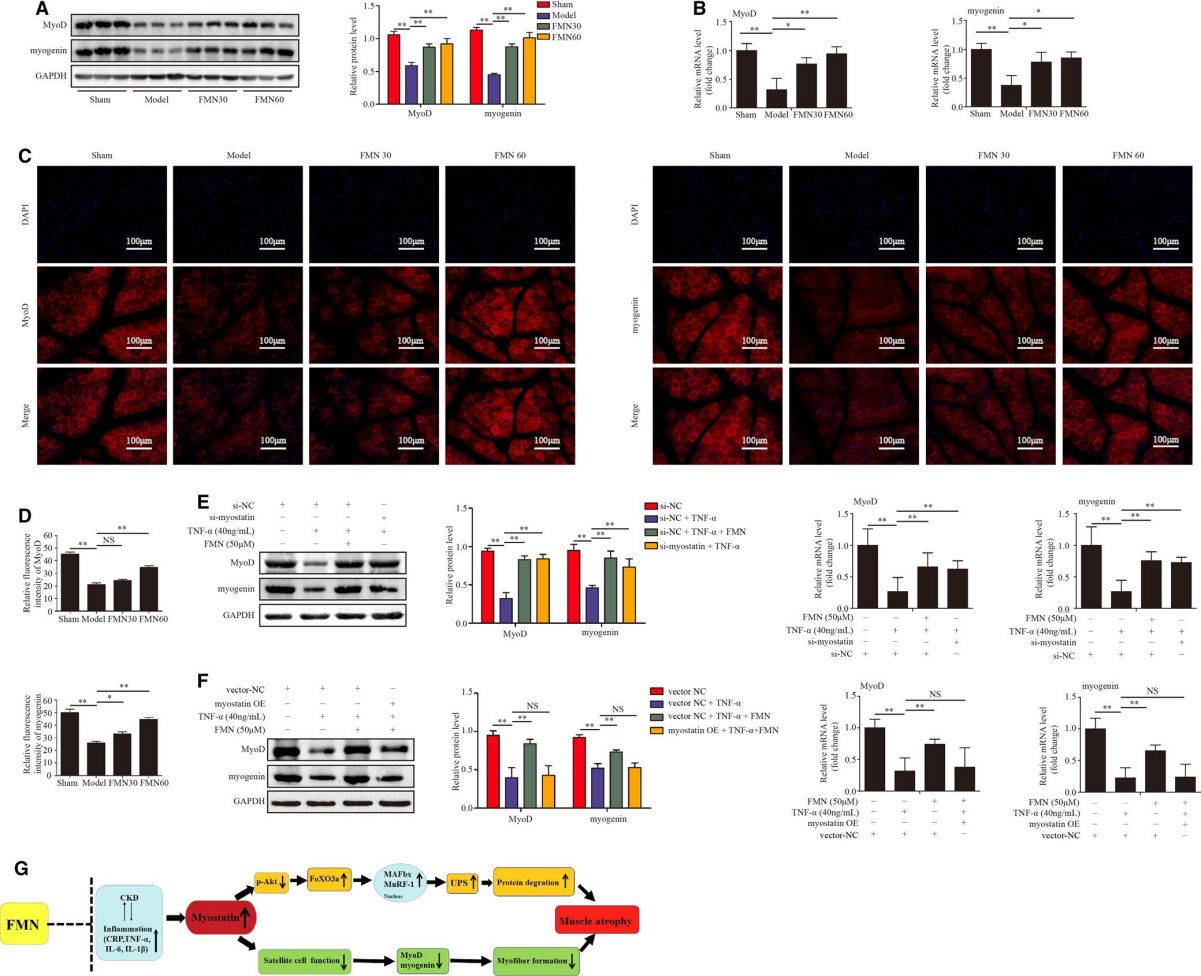
FMN improves satellite cell proliferation and differentiation in CKD rats and C2C12 myotubes. (A) Protein levels of MyoD and myogenin in gastrocnemius muscle of different groups analysed by Western blotting (n = 3/group). (B) mRNA levels of MyoD, myogenin in gastrocnemius muscle analysed with qPCR (n = 3/group). (C) Sections of tibialis anterior muscle from different groups were determined with immunofluorescence staining (200×) with anti‐MyoD, anti‐myogenin (red) and DAPI (blue). (D) The relative fluorescence intensity of MyoD and myogenin was compared between groups. (E) C2C12 myotubes were treated with FMN (50 μmol/L) in the presence or absence of TNF‐α (40 ng/mL) for 48 h following 24 h of incubation with si‐myostatin or si‐NC. The protein and mRNA levels of MyoD and myogenin in C2C12 myotubes using Western blotting and qPCR (n = 3/group). (F) Myostatin OE transfection was used to overexpress myostatin in C2C12 myotubes, and then, they were incubated with FMN (50 μmol/L) and TNF‐α for another 48 h. The protein and mRNA levels of MyoD and myogenin in C2C12 myotubes were assessed with Western blotting and qPCR. All values are presented as means ± SDs for three independent experiments (n = 3/group). *P < .05, **P < .01. (G) Summative diagram of FMN alleviating CKD muscle atrophy

The effects of FMN on the function of satellite cells were detected through measurement of the expressions of MyoD and myogenin in the muscles of CKD rats. Western blotting and qPCR results showed that the levels of MyoD and myogenin were significantly lower in the muscles of CKD model group than in the sham group, but this was ameliorated by FMN (Figure 6A,B). The immunofluorescence staining results of MyoD and myogenin in the tibialis anterior muscle showed a similar trend (Figure 6C,D).

In vitro, to verify the effect of FMN on myogenic differentiation, the expressions of MyoD and myogenin in TNF‐α‐induced C2C12 myotubes were detected. Western blotting and qPCR showed that TNF‐α significantly suppressed the expressions of MyoD and myogenin, while FMN (50 μmol/L) and si‐myostatin attenuated this inhibition (Figure 6E). However, myostatin OE significantly blocked the change in the expressions of MyoD and myogenin by FMN in C2C12 myotubes (Figure 6F).

## 4 DISCUSSION

Muscle wasting is a frequent and serious complication of CKD, and it is correlated with risk for morbidity and mortality in this condition. No clinically reliable methods of preventing CKD muscle atrophy currently exist. Therefore, study of its molecular mechanisms is urgently needed. New treatment methods and treatment strategies must be sought. Our findings indicate that inflammation and myostatin are activated in CKD‐induced muscle atrophy. FMN has an anti‐inflammatory effect and inhibition of myostatin expression in CKD muscle atrophy. It also attenuates muscle atrophy by improving the myostatin‐mediated dephosphorylation of PI3K/Akt/FoxO3a pathway and the impaired satellite cell proliferation and differentiation.

Inflammation plays an important role in the pathogenesis of CKD muscle atrophy.^34^ Previous studies support a direct pathological role for CRP, TNF‐α, IL‐6 and IL‐1β in CKD muscle atrophy.^3^, ^20^ Elevated levels of CRP produce acute inflammatory responses, which is a notable performance in CKD and serves as an important marker for stratifying cardiovascular risk in them.^4^, ^35^ The infusion of TNF‐α, IL‐6 and IL‐1β increased the degradation of muscle protein, but the reduction of these inflammatory cytokines with pharmacological methods attenuated muscle atrophy.^36^, ^37^ In our study, we found that the inflammatory markers CRP, TNF‐α, IL‐6 and IL‐1β in serum and muscles were markedly increased in CKD rats, which is consistent with previous reports.^7^, ^12^ In addition, we established a muscle atrophy model in vitro in TNF‐α‐induced C2C12 myotubes. Immunofluorescence staining showed that TNF‐α up‐regulated the expressions of the skeletal muscle atrophy‐specific genes MAFbx and MuRF‐1 and increased the levels of inflammatory markers. FMN significantly reduced the levels of the inflammatory markers in CKD rats and TNF‐α‐induced C2C12 myotubes. These results reveal that inflammation is involved in CKD muscle atrophy, but FMN has a countering anti‐inflammatory effect.

Myostatin is a proverbial negative regulator of muscle mass in CKD muscle atrophy. Abundant evidence has indicated that myostatin expression is increased in the muscles of CKD patients.^11^, ^13^, ^24^ Myostatin is related to the up‐regulation of inflammatory cytokines, particularly TNF‐α. In a mouse model of CKD, TNF‐α increased the expression of myostatin via the NF‐κB pathway to accelerate the process of muscle atrophy.^38^ NF‐κB activation is required for cytokine‐induced skeletal muscle protein loss in CKD patients.^12^, ^13^, ^39^ Consistent with previous studies, the expression of mRNA and the protein levels of myostatin were increased in the muscles of CKD rats. Meanwhile, we also observed that TNF‐α increased the expression of myostatin in C2C12 myotubes. Moreover, FMN decreased the expression of myostatin in the CKD rats and the TNF‐α‐induced C2C12 myotubes. si‐myostatin also reduced myostatin expression in TNF‐α‐induced C2C12 myotubes. These results indicate that inflammation may activate the expression of myostatin, and FMN may inhibit the expression of myostatin in CKD rats and TNF‐α‐induced C2C12 myotubes.

The mechanisms of myostatin‐induced muscle atrophy in CKD include the stimulation of protein degradation. The insulin /IGF‐1 signalling pathway is the key to regulate the muscle protein metabolism. This pathway is activated when the phosphorylation of intermediate mediators of insulin/IGF‐1 signalling, such as PI3K and Akt. The phosphorylation of Akt regulates protein metabolism by increasing protein synthesis and inhibiting protein degradation.^8^ In CKD, the inhibition of the insulin/IGF‐1 signal results in a decrease in PI3K activity, which also results in a decrease in Akt phosphorylation. Low‐levels of phosphorylated Akt also down‐regulate the phosphorylation of the Forkhead box O (FoxO) family of transcription factors, such as FoxO3a. Activated (dephosphorylated) FoxO enters the nucleus to increase the expressions of the muscle‐specific E3 ubiquitin ligases MuRF1 and MAFbx to activate UPS and cause muscle atrophy.^4^, ^40^, ^41^, ^42^ MAFbx and MuRF1 are the key enzymes leading to skeletal muscle protein degradation and are excellent markers of muscle atrophy. Both MuRF1 and MAFbx are atrogenes, or atrophy‐related genes, and mainly expressed in muscle tissues. Under a variety of muscle atrophy conditions, the expressions of MuRF1 and MAFbx are significantly increased.^43^ They are involved in the activation of the UPS pathway, which is the major mechanism for the protein degradation in muscle. The present study found that CKD significantly increases the expressions of MAFbx and MuRF‐1 and decreases the expressions of p‐PI3K, p‐Akt and p‐FoxO3a associated with increased muscle protein degradation in CKD rats. Notably, FMN inhibited the expressions of MAFbx and MuRF‐1 and up‐regulated the expressions of p‐PI3K, p‐Akt and p‐FoxO3a. In addition, the effects of FMN were further verified in TNF‐α‐induced C2C12 myotubes. TNF‐α increased the expressions of both MAFbx and MuRF‐1 and decreased the expressions of p‐PI3K, p‐Akt and p‐FoxO3a. FMN and si‐myostatin inhibited the expressions of MAFbx and MuRF‐1 and up‐regulated the phosphorylation of PI3K, Akt and FoxO3a induced by TNF‐α in C2C12 myotubes. These results indicate that FMN inhibits the activation of UPS and up‐regulates the phosphorylation of the PI3K/Akt/FoxO3a pathway in the muscle of CKD rats and TNF‐α‐induced C2C12 myotubes.

In addition, myostatin induces muscle atrophy by inhibiting the satellite cell functions of proliferation and differentiation. Mature muscles are composed of muscle fibres surrounded by the basal lamina, which is not only covered with myofibrils, but also with satellite cells.^44^ Satellite cells, known as muscle precursor cells, are usually quiescent, but when the muscle fibres are damaged, the satellite cells attached to the surface of the muscle fibres are activated and expressed myogenic markers of proliferation and differentiation, including MyoD and myogenin. Satellite cells can repair injured muscle fibres or lead to the formation of new ones. MyoD and myogenin are the master switches for satellite cell activation. In a previous study that employed a mouse model of CKD, the expressions of MyoD and myogenin released from satellite cells were reduced, which was associated with satellite cell dysfunction.^45^ Our results indicate that CKD significantly inhibited the expressions of MyoD and myogenin correlated with the function of satellite cells, which is consistent with previous reports. Notably, FMN improved this impaired function in the muscles of CKD rats. We examined these effects in C2C12 myotubes, and the results confirmed the in vivo experiments.

To further verify the improvement effects and related mechanisms of FMN on CKD muscle atrophy, myostatin OE was used to transiently transfect C2C12 myotubes. As expected, the effects of FMN on UPS and the PI3K/AKT/FoxO3a signalling pathway were compromised, as were the proliferation and differentiation function of the C2C12 myoblasts. Above all, we found that FMN attenuates myostatin‐mediated muscle atrophy by up‐regulating the phosphorylation of the PI3K/Akt/FoxO3a pathway and improving the satellite cells dysfunction.

In conclusion, our findings indicate that FMN has an anti‐inflammatory effect in CKD‐induced muscle atrophy and TNF‐α‐induced C2C12 myotubes. It attenuates muscle atrophy by improving myostatin‐mediated dephosphorylation of the PI3K/Akt/FoxO3a pathway and the impaired proliferation and differentiation functions of satellite cells. These findings highlight the potential application of FMN to the treatment of muscle atrophy in CKD patients.

## CONFLICT OF INTEREST

The authors declare that the research has no conflicts of interest.

## AUTHOR CONTRIBUTIONS

**Lingyu Liu:** Methodology (lead); Software (lead); writing‐original draft (lead); writing‐review & editing (lead). **Rong Hu:** Data curation (equal); software (supporting). **Haiyan You:** Methodology (lead); resources (supporting). **Jingjing Li:** Data curation (supporting); software (supporting). **Yangyang Liu:** Resources (supporting). **Qiang Li:** Methodology (supporting). **Xiaohui Wu:** Software (supporting). **Jiawen Huang:** Data curation (supporting). **Xiangsheng Cai:** Investigation (supporting); supervision (supporting). **Mingqing Wang:** Investigation (supporting); supervision (supporting). **Lianbo Wei:** Funding acquisition (lead); project administration (lead); supervision (lead).

## Data Availability

All the data supporting these results are shown in the paper and are available from the corresponding authors.
